# Risk factors associated with knee osteoarthritis severity and their correlation with Kellgren–Lawrence grade

**DOI:** 10.3389/fmed.2025.1744478

**Published:** 2026-01-13

**Authors:** Taiya Chen, Jie Ma, Bi Liu, Shelei Huang

**Affiliations:** 1 Department of Radiology, Shenzhen People's Hospital (The Second Clinical Medical College of Jinan University), Shenzhen, Guangdong, China; 2 The First Affiliated Hospital, Southern University of Science and Technology, Shenzhen, Guangdong, China; 3 Department of Orthopaedics, Shenzhen People's Hospital (The Second Clinical Medical College of Jinan University), Shenzhen, Guangdong, China

**Keywords:** body mass index, C-reactive protein, inflammation, Kellgren–Lawrence grade, knee osteoarthritis, pain measurement, risk factors, severity

## Abstract

**Objective:**

To identify the risk factors associated with knee osteoarthritis (KOA) radiographic severity in a defined cohort of patients with KOA and to examine their associations with Kellgren–Lawrence (K–L) grade.

**Methods:**

In this retrospective study, 258 patients with confirmed KOA (100 males and 158 females) diagnosed between July and November 2024 at Shenzhen People’s Hospital were enrolled. Demographic and clinical data—including age, sex, body-mass index (BMI), K–L grade, C-reactive protein (CRP) level, and pain intensity assessed by the visual analogue scale (VAS)—were extracted. Sex-related differences in age, K–L grade, BMI, CRP, and VAS were analyzed using the Mann–Whitney *U*-test, Chi-square test, and independent t-test. Spearman rank correlation was employed to evaluate the associations between each variable and K–L grade within each sex. Multivariable logistic regression was performed to examine the associations of each factor with mild (K–L 1–2) versus moderate-to-severe (K–L 3–4) KOA. ROC curves were constructed to assess the discriminative accuracy for moderate-to-severe K–L grade.

**Results:**

(1) Significant gender differences were observed in height, weight, and K–L grade distribution among KOA patients (all *P* < 0.05). In contrast, age, BMI, CRP levels, and VAS pain scores showed no statistically significant gender variations (all *P* > 0.05). (2) CRP exhibited the strongest correlation with K–L grade in both sexes (*r* = 0.51 in males, *r* = 0.60 in females). (3) Multivariable logistic regression analysis, after adjusting for age, BMI, and VAS pain score, revealed that CRP showed the strongest association with K–L grade among the factors in both sexes. (4) ROC analysis revealed good discriminatory performance for the combined model in distinguishing mild from moderate-to-severe KOA, with AUC = 0.865 (95% CI: 0.793–0.938) in males and AUC = 0.880 (95% CI: 0.827–0.933) in females, indicating good discriminatory performance within this dataset. It should be noted that these results reflect the model’s performance in the current sample and internal validation would be needed to assess generalizability.

**Conclusion:**

In this cross-sectional study, CRP showed a strong association with KOA radiographic severity after adjusting for age, BMI, and pain scores. A numerically stronger association was observed in female patients, though this difference requires further confirmation. These findings highlight the potential role of systemic inflammation in KOA and support the further investigation of CRP as a supplementary clinical assessment tool.

## Introduction

Knee osteoarthritis (KOA) is a chronic, degenerative disorder characterised by progressive articular cartilage loss, osteophyte formation, and pathological changes in peri-articular tissues. It represents one of the most prevalent degenerative joint diseases and typically manifests as joint pain, swelling, and impaired mobility (1, 2). With global population ageing, arthritis has become a leading cause of reduced mobility and disability among middle-aged and older adults (3, 4).

Established risk factors for disease severity include obesity, history of joint trauma, advanced age, and female sex (5, 6). More recently, mounting evidence has implicated inflammatory biomarkers—C-reactive protein (CRP) and its metabolite CRPM (C-reactive protein metabolite, CRPM), which is released from inflamed tissue following degradation by matrix metalloproteinases (MMPs) such as those secreted by macrophages—as additional determinants of KOA severity (7–9). Furthermore, pain is also recognized as a primary driver of clinical decision-making and the utilization of healthcare resources (2). Kellgren–Lawrence (K–L) grading, the radiographic gold standard for assessing KOA severity, demonstrates marked inter-individual variability in severity (10). The coexistence of these heterogeneous and multifactorial elements underscores the complex and multifaceted nature of KOA severity (11). Such complexity may yield discordant symptomatic and structural phenotypes between men and women, potentially leading to divergent disease trajectories.

Contemporary KOA research increasingly emphasises patient stratification strategies that acknowledge disease heterogeneity and the need for personalised therapeutic approaches. These strategies seek to classify patients according to their risk-factor profiles, thereby moving beyond traditional “one-size-fits-all” management paradigms (12). Consequently, elucidating sex-specific differences in factors influencing KOA severity has become imperative. Against the backdrop of population ageing and the era of precision medicine, a nuanced understanding of sex-related disparities in KOA carries significant public-health and clinical value.

While several systematic reviews have summarized risk factors for KOA progression, few have directly compared the sex-specific associative strength of inflammatory markers (e.g., CRP) with traditional factors (e.g., BMI and age) in a clinical Asian cohort. Furthermore, sex-specific clinical features in the association between CRP and radiographic severity remain insufficiently explored.

Guided by these clinical observations and scientific insights, the present study was designed to address three key objectives. First, we aimed to comprehensively characterise the sex-specific features distributions of K–L grade, CRP level, BMI, and pain intensity among a defined cohort of patients with KOA. We will evaluate the interplay among age, BMI, CRP, VAS score, and KOA severity to uncover potential sex-specific clinical features. Second, multivariable regression analyses will be employed to examine the associations of these factors with K–L grade (as a measure of radiographic severity) in male and female patients separately. Finally, sex-stratified diagnostic models will be developed to discriminate between mild and moderate-to-severe KOA at presentation, which could aid clinicians in patient stratification and initial management planning.

## Methods

### Study design and participants

This retrospective study consecutively enrolled patients with a confirmed diagnosis of knee osteoarthritis (KOA) presenting to the Department of Orthopaedics, Shenzhen People’s Hospital, between July and November 2024. Given that all patients enrolled in this study were of Chinese nationality, the diagnostic criteria for patient recruitment were formulated in accordance with the 2021 Guidelines for the Diagnosis and Treatment of Osteoarthritis in China (13), All patients were independently diagnosed by orthopedic specialists based on these criteria, which require the presence of knee pain plus at least three of the following: age ≥50 years, morning stiffness <30 min, crepitus, bony tenderness, bony enlargement, or no palpable warmth.

#### Inclusion criteria

(1) Age ≥ 18 years. While knee osteoarthritis is typically age-related, the inclusion of adults aged 18 years and older allows for the assessment of early-onset or atypical cases. All enrolled patients met the diagnostic criteria for osteoarthritis per the 2021 Chinese Guidelines, ensuring that juvenile arthritis or inflammatory arthritides were excluded. (2) Definitive KOA diagnosis by an orthopaedic specialist. (3) Interval between serum C-reactive protein (CRP) measurement and weight-bearing anteroposterior knee radiography ≤7 days.

#### Exclusion criteria

(1) Recent acute infection or any systemic inflammatory disorder (e.g., rheumatoid arthritis, gouty arthritis, systemic lupus erythematosus). (2) Severe cardiovascular disease, uncontrolled diabetes mellitus, or any other condition known to significantly influence CRP levels. (3) History of knee surgery or any major surgical procedure within the preceding 3 months. (4) Patients who were using anti-inflammatory medications (including NSAIDs, corticosteroids, or DMARDs) within 4 weeks prior to CRP measurement, or who had received rehabilitative interventions (e.g., dry needling, cupping, intensive manual therapy) within 7 days before assessment, were excluded to minimize confounding effects on CRP and pain scores.

Demographic and clinical data—including age, sex, height, weight, body mass index (BMI), Kellgren–Lawrence (K–L) grade, visual analogue scale (VAS) pain score at admission (14), and serum CRP concentration—were extracted from the electronic medical record system. Height and weight were measured by trained nursing staff using standardized stadiometers and scales during hospital admission.

### Radiographic assessment of KOA severity

Knee radiographs were graded using the Kellgren–Lawrence (K–L) classification (15):

Grade 1: doubtful joint-space narrowing and possible osteophytic lipping.

Grade 2: definite osteophytes and possible joint-space narrowing.

Grade 3: moderate joint-space narrowing, definite osteophytes, some sclerosis, and possible bony deformity.

**Figure 1:**
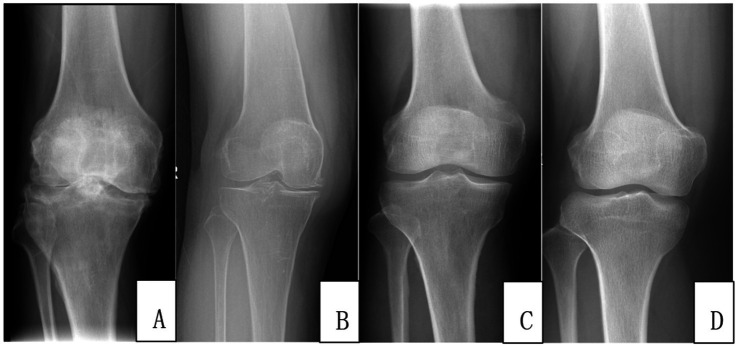
Kellgren–Lawrence (K–L) grade and C-reactive protein (CRP) level in the right knee joint among patients of different ages. Patient **(A)**, K–L grade 4, CRP 43.5 mg/L; patient **(B)**, K–L grade 3, CRP 20.8 mg/L; patient **(C)**, K–L grade 2, CRP 8.6 mg/L; patient **(D)**, K–L grade 1, CRP 3.0 mg/L.

Grade 4: large osteophytes, marked joint-space narrowing, severe sclerosis, and definite bony deformity (Figure 1).

Weight-bearing anteroposterior (AP) and lateral radiographs of both knees were obtained with the patient standing in full extension, using a standardized imaging protocol. Two radiologists—one attending radiologist with >10 years of experience and one resident with 3 years of experience—performed all grading in blinded fashion, without knowledge of clinical data. The higher grade of both knees was recorded; discrepancies were resolved by consensus. Consistent with Kim et al. (16), K–L grades 1–2 were classified as mild, and grades 3–4 as moderate-to-severe KOA.

### BMI classification

Following World Health Organization criteria, BMI ≥ 25 kg/m^2^ was defined as overweight, and BMI ≥ 30 kg/m^2^ as obese.

### Visual analogue scale (VAS) for pain

Pain intensity was quantified using the 10-cm VAS, anchored at 0 cm (“no pain”) and 10 cm (“worst imaginable pain”). Patients marked the point corresponding to their current pain level. The VAS is a validated, highly sensitive instrument for acute and chronic pain assessment.

### Inflammatory biomarker: CRP

Serum CRP was measured by immunoturbidimetry on a Beckman Coulter analyser. Blood samples were collected via standard venepuncture and processed according to manufacturer protocols. The institutional reference range for CRP is <5 mg/L.

### Sample size consideration and sampling technique

A consecutive sampling method was adopted in this study to minimize selection bias. Given that this study is a retrospective observational study, the sample size was naturally determined by the total number of patients who met all pre-specified inclusion and exclusion criteria and had complete data during the study period (July to November 2024). To evaluate whether the current sample size is sufficient for the planned multivariable logistic regression analysis, we performed a post-hoc assessment using the “events per variable (EPV)” criterion, which is critical for ensuring model stability and avoiding overfitting. The outcome variable was dichotomized into mild knee osteoarthritis (K–L grades 1–2) and moderate-to-severe knee osteoarthritis (K–L grades 3–4). In the male cohort, there were 61 events (i.e., patients with moderate-to-severe KOA), and in the female cohort, there were 117 events. Each gender-stratified logistic regression model included 4 predictor variables (age, BMI, CRP, and VAS pain score). Accordingly, the calculated EPV values were 15.25 in males and 29.25 in females. An EPV value of ≥10 is generally recommended to ensure the reliability of parameter estimation. The EPV values in this study were significantly greater than 10, indicating that the current sample size is sufficient to support the conducted multivariable analyses.

### Statistical analysis

All analyses were performed using SPSS version 22.0 for Windows (IBM Corp.). Continuous variables are presented as mean ± standard deviation (SD) or median with interquartile range (IQR), depending on distribution. Normality was assessed with the Kolmogorov–Smirnov test. Sex-related differences in age, K–L grade, BMI, CRP, and VAS were analysed using the Mann–Whitney U test, Chi-square test and independent t-test. Spearman rank correlation was used to examine the associations among CRP, BMI, age, and K–L grade; corresponding heat maps were generated.

Preliminary analyses indicated that sex may exert an effect modification. Particularly, this may be attributable to the impact of endogenous hormones in females. Therefore, we constructed separate multivariable logistic regression models for males and females to explore the sex-specific associations between predictors and the Kellgren–Lawrence (K–L) grade. The outcome variable was dichotomized as 0 for mild KOA (K–L grades 1–2) and 1 for moderate-to-severe KOA (K–L grades 3–4). The relative strength of association of individual factors was derived from standardized regression coefficients. Receiver-operating characteristic (ROC) curves were plotted for each sex. The combined model included age, BMI, CRP, and VAS. Statistical significance was set at *p* < 0.05 (two-tailed).

## Results

### Demographic and clinical characteristics

After application of inclusion and exclusion criteria, 258 patients (100 men, 158 women) were enrolled. Ages ranged from 26 to 97 years in men and from 29 to 96 years in women. Four patients (3 men, 1 woman) were under 35 years old; all had a history of significant knee trauma. Data on genetic forms of OA were not systematically collected. K–L grade distribution was as follows: grade 1, 9 (3.5%); grade 2, 71 (27.5%); grade 3, 112 (43.4%); grade 4, 66 (25.6%).

### Between-sex comparisons

Significant gender differences were observed in height, weight, and K–L grade distribution among KOA patients (all *P* < 0.05). In contrast, age, BMI, CRP levels, and VAS pain scores showed no statistically significant gender variations (all *P* > 0.05) (Table 1).

**Table 1:** Between-sex comparison of clinical variables in patients with KOA.

Variable	Men	Women	*Z*/*χ*^2^/*t*	*P*
Age (years)	66.33 ± 13.81	65.22 ± 11.01	−1.32	0.187
K–L grade, *n* (%)			19.04	0.003^*^
Grade 1	9 (9.0%)	0 (0%)		
Grade 2	30 (30.0%)	41 (25.9%)		
Grade 3	33 (33.0%)	79 (50.0%)		
Grade 4	28 (28.0%)	38 (24.1%)		
CRP (mg/L)	3.35 (1.40, 11.28)	3.45 (1.30,9.13)	−0.59	0.554^#^
VAS (points)	2.00 (1.00, 2.00)	2.00 (1.00,2.00)	−0.20	0.840^#^
Height (cm)	167.80 ± 7.08	156.40 ± 10.19	−11.22	<0.001
Weight (kg)	72.57 ± 12.74	62.70 ± 9.81	−6.51	<0.001
BMI (kg/m^2^)	25.72 ± 3.74	25.62 ± 3.69	−0.24	0.808

Data are presented as mean ± SD, median [IQR], or n (%).

^*^Chi-square test for categorical variables.

^#^Mann–Whitney U test for non-normally distributed variables (CRP, VAS).

Other continuous variables were compared using independent t-test.

Statistical significance was set at *p* < 0.05 (two-tailed).

BMI, body mass index; CRP, C-reactive protein; IQR, interquartile range; K–L, Kellgren–Lawrence; VAS, visual analogue scale.

### Correlations between variables and K–L grade

**Figure 2:**
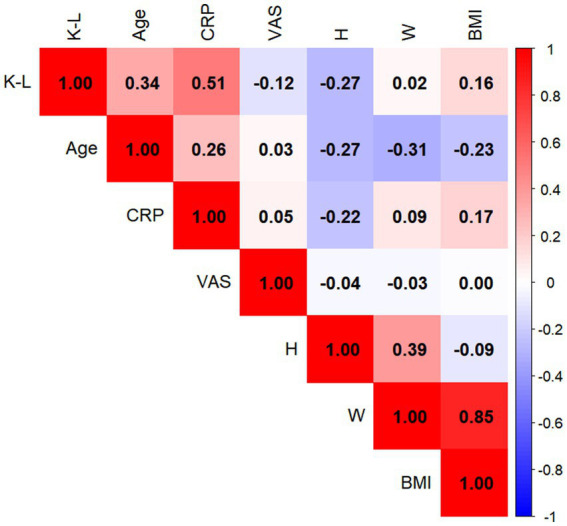
Heatmap illustrating the correlations between various factors and the Kellgren–Lawrence (K–L) grade of the knee joint in male patients with knee osteoarthritis. Color intensity reflects the magnitude of the Spearman correlation coefficient (*r*); red denotes positive correlations, whereas blue denotes negative correlations. C-reactive protein exhibited the strongest positive correlation with K–L grade (*r* = 0.51, *P* < 0.05).

**Figure 3:**
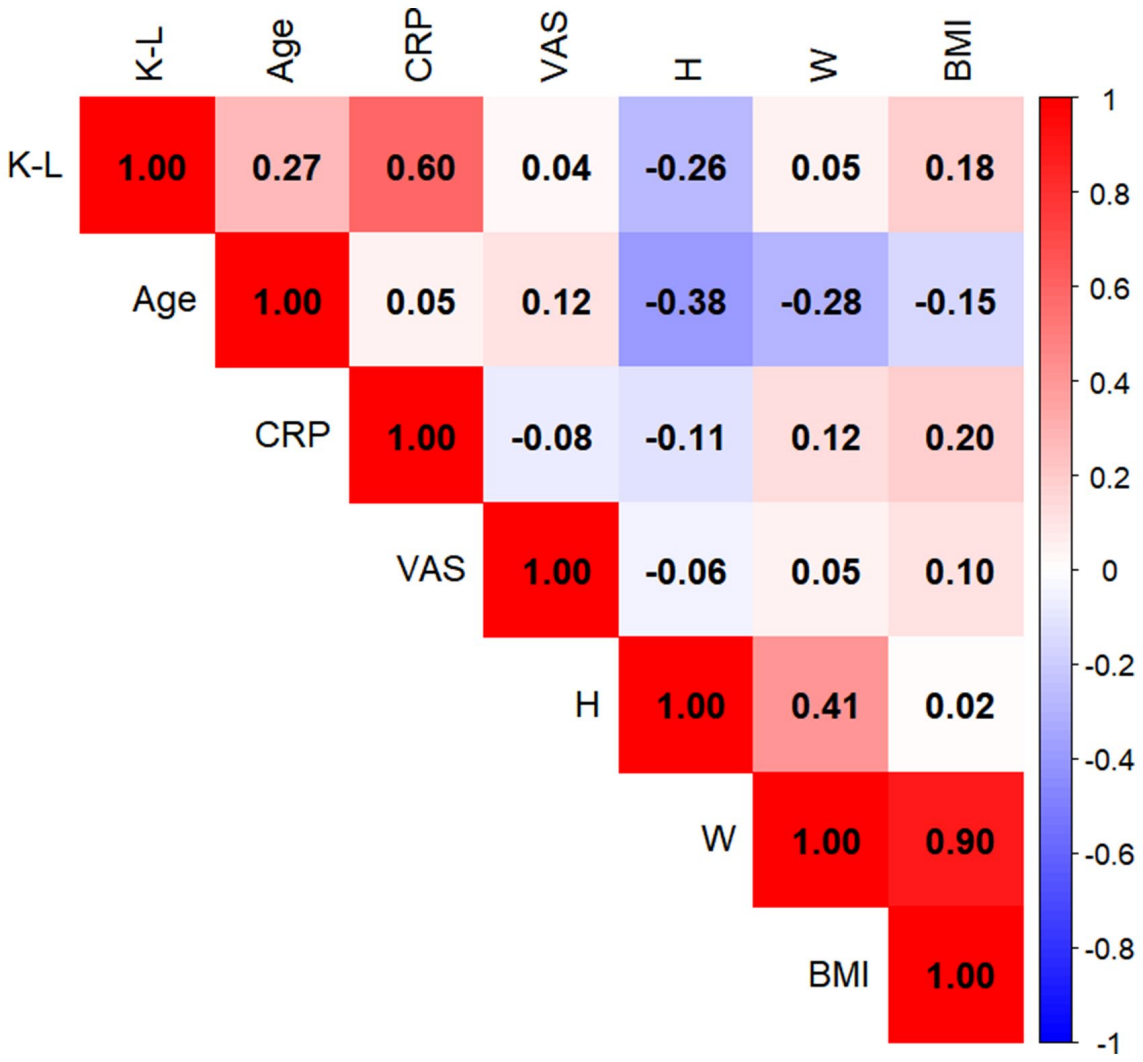
Heatmap depicting the correlations between various factors and the Kellgren–Lawrence (K–L) grade of the knee joint in female patients with knee osteoarthritis. Color intensity corresponds to the magnitude of the Spearman correlation coefficient (*r*); red indicates positive correlations, whereas blue indicates negative correlations. C-reactive protein demonstrated the strongest positive correlation with K–L grade (*r* = 0.60, *P* < 0.05).

In men, CRP exhibited the strongest correlation with K–L grade (*r* = 0.51, *p* < 0.05); in women, CRP also demonstrated the strongest correlation (*r* = 0.60, *p* < 0.05) (Figures 2, 3).

### Logistic regression analysis

**Figure 4:**
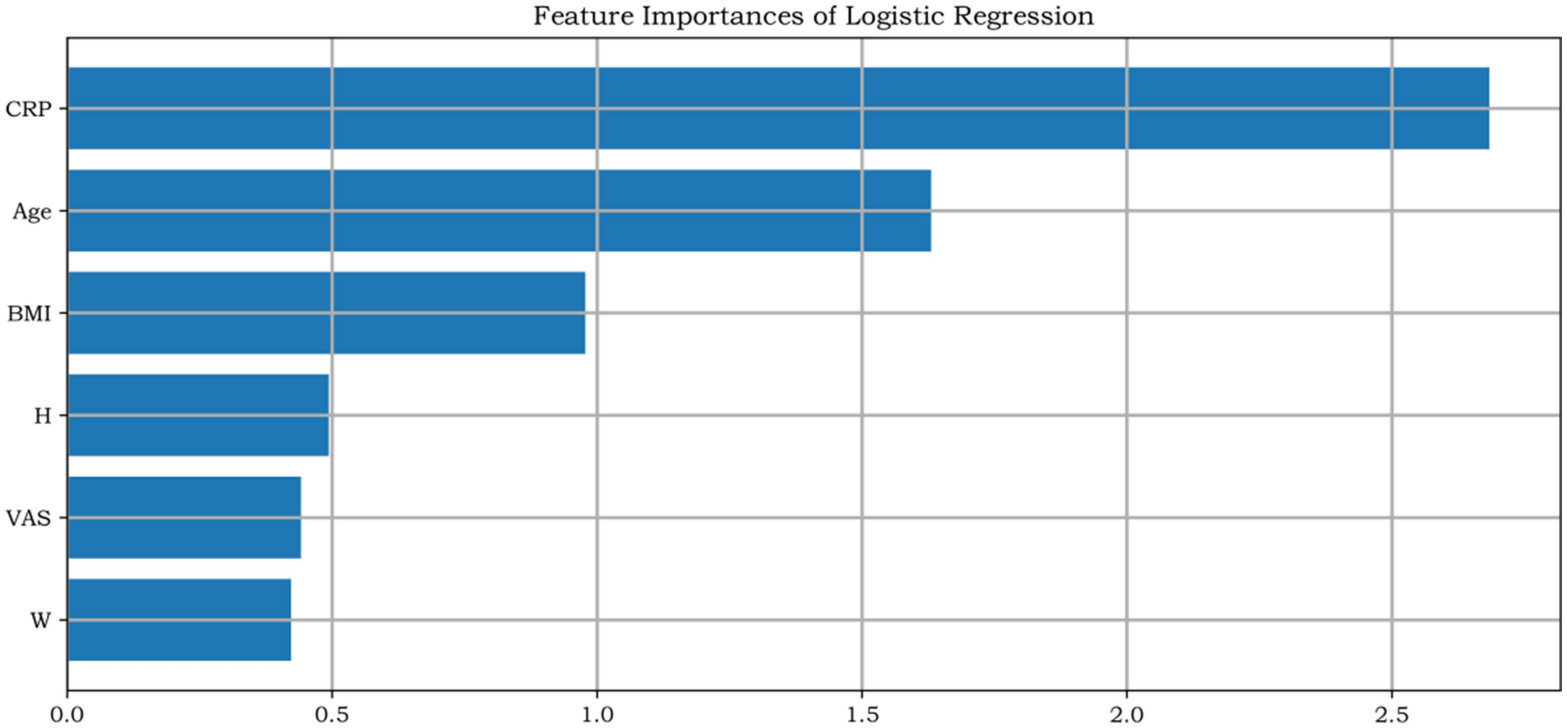
Relative contribution weights of individual factors to Kellgren–Lawrence (K–L) grade in male patients with knee osteoarthritis.

**Figure 5:**
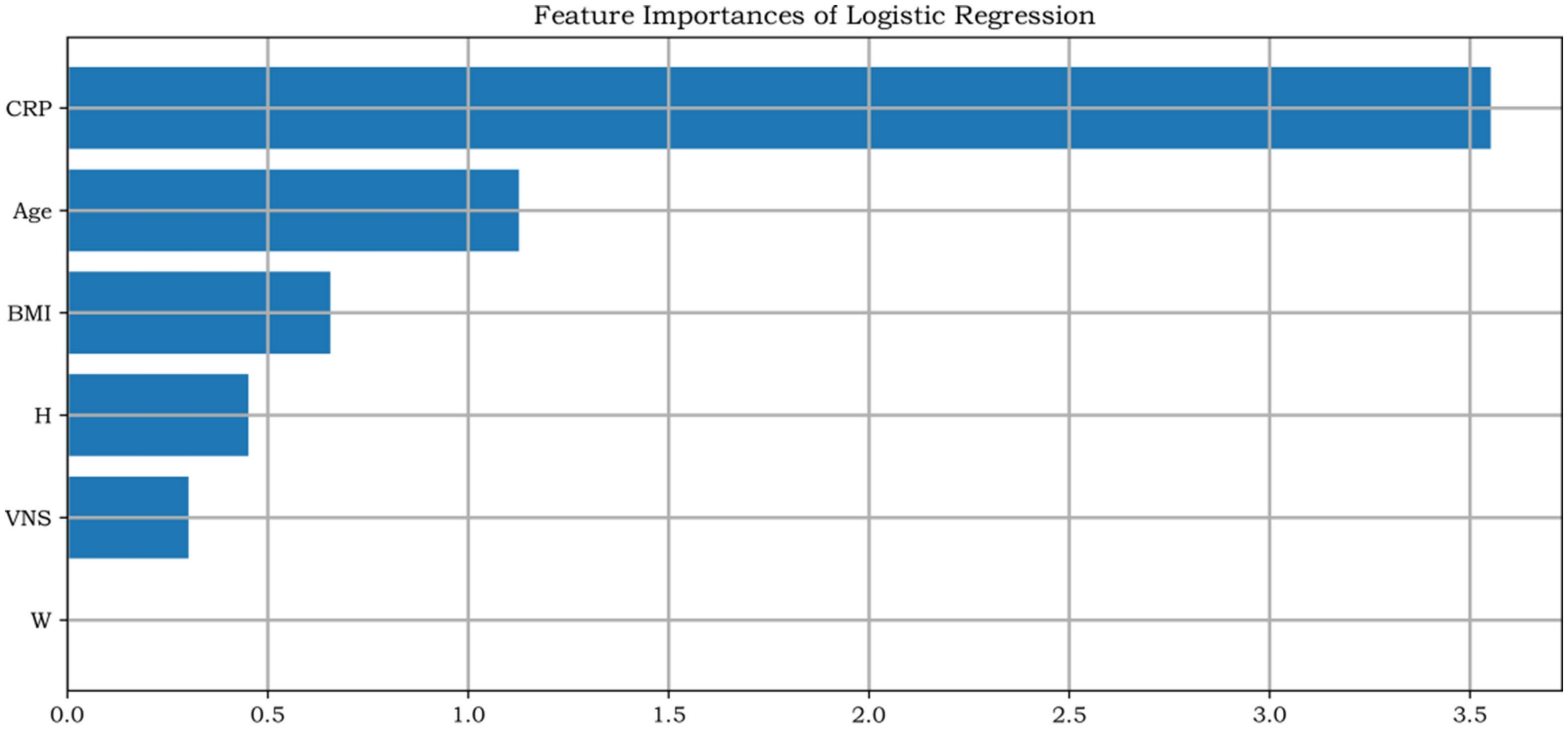
Relative contribution weights of individual factors to Kellgren-Lawrence (K-L) grade in female patients with knee osteoarthritis.

Multivariable logistic regression revealed that, in both sexes, CRP showed the strongest association with K–L grade among the predictors in the model after adjusting for age, BMI, and VAS pain score (Figures 4, 5). The standardized regression coefficients, Odds Ratios (OR), 95% Confidence Intervals (CI), and *p*-values are presented in Table 2.

**Table 2:** Sex-stratified multivariable logistic regression analysis of factors associated with moderate-to-severe knee osteoarthritis (K–L grades 3–4).

Sex	Risk factors	Standardized regression coefficient (*β*)	OR	95% confidence interval (CI)	*P*-value
Male (*n* = 100)	Age	0.213	1.032	1.001–1.064	0.042
	BMI	0.187	1.087	0.985–1.201	0.105
	CRP	0.468	1.124	1.068–1.183	<0.001
	VAS	0.042	1.056	0.823–1.356	0.663
Female (*n* = 158)	Age	0.257	1.041	1.015–1.068	0.002
	BMI	0.221	1.103	1.021–1.192	0.014
	CRP	0.532	1.157	1.109–1.207	<0.001
	VAS	0.076	1.089	0.901–1.318	0.397

Outcome variable: Kellgren–Lawrence grade; Mild = grades 1–2, Moderate-to-severe = grades 3–4.

### ROC analysis

**Figure 6:**
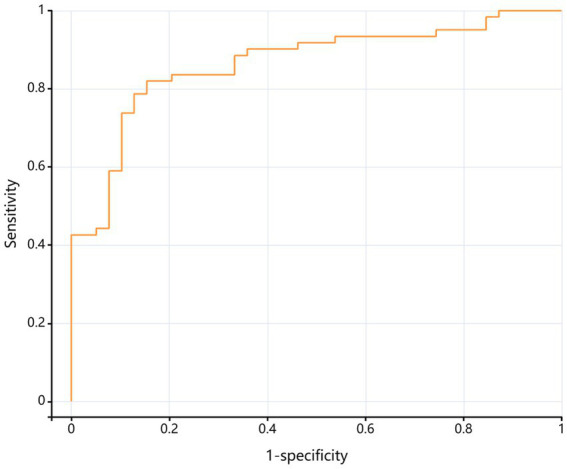
ROC curve for the discrimination of moderate-to-severe K–L grade in male KOA patients.

**Figure 7:**
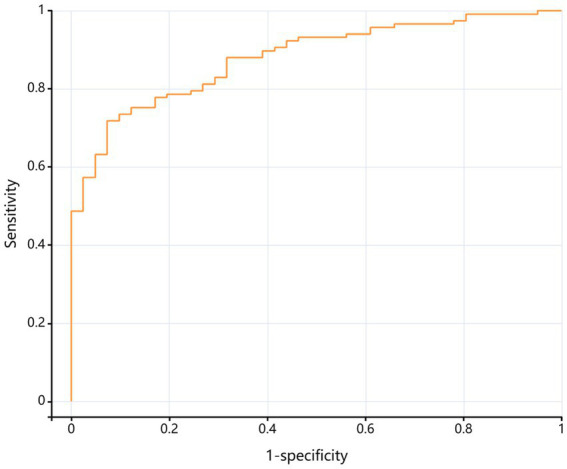
ROC curve for the discrimination of moderate-to-severe K-L grade in female KOA patients.

The combined model (including age, BMI, CRP, and VAS) achieved an AUC of 0.865 (95% CI 0.793–0.938) in men (Figure 6) and 0.880 (95% CI 0.827–0.933) in women (Figure 7), indicating excellent discriminatory performance for distinguishing mild from moderate-to-severe KOA.

## Discussion

In this retrospective analysis, we systematically evaluated the clinical characteristics of patients with knee osteoarthritis (KOA) and found that C-reactive protein (CRP) was more strongly associated with radiographic severity than traditional factors such as body mass index (BMI) and age, with a notably stronger correlation in female patients (*r* = 0.60 vs. *r* = 0.51 in males). However, this difference should be interpreted cautiously as a formal statistical comparison was not performed, and the sample sizes differed between groups. Although previous investigations have extensively examined the influence of BMI and age on KOA, our study suggests that inflammatory biomarkers specifically CRP may have stronger associations with disease severity than previously recognized. This finding offers a readily accessible biomarker for clinical assessment and reinforces the role of inflammatory mechanisms in KOA. Furthermore, using a sex-stratified approach, we demonstrated that CRP levels correlated moderately with Kellgren–Lawrence (K–L) grade, and this correlation was numerically stronger in women. Finally, we constructed receiver operating characteristic (ROC) curves to assess the discriminative ability of CRP combined with traditional variables for moderate-to-severe K–L grade in both sexes, confirming the good discriminatory performance of this combined model within the current dataset. However, since these results are based on the same dataset used to develop the model, internal or external validation would be needed to confirm its generalizability.

A total of 258 patients diagnosed with KOA (100 males and 158 females) were enrolled. Age, BMI, visual analogue scale (VAS) score, and CRP level showed no statistically significant intersex differences (*p* > 0.05). Several explanations may account for this observation. First, baseline characteristics including age distribution and BMI were well matched between sexes, potentially attenuating any underlying sex-related disparities. Second, the assessment tools employed (e.g., VAS) may exhibit limited sensitivity to subtle or early sex-specific differences. These findings underscore the multifactorial complexity of KOA, wherein disease severity mechanisms may exhibit greater similarities than differences between sexes, particularly when baseline characteristics are comparable. Similar studies have reported that sex disparities may be diminished under conditions of balanced baseline features (6).

Our observation of comparable VAS scores between sexes aligns with the findings of Racine et al. (17); however, prior evidence has suggested that women may experience more severe pain (18). The similarity in pain ratings observed in the current study may reflect reporting bias inherent in subjective assessments or limitations of the pain-evaluation instruments. Huskisson (14) noted that, although widely utilised, the VAS can be influenced by cultural, psychological, and emotional factors. Moreover, Osborne and colleagues (19) emphasised that pain is a subjective, multidimensional experience encompassing sensory, affective, and cognitive components; consequently, sex may modulate pain perception through multiple pathways.

Obesity is a recognised risk factor for KOA development, yet no significant sex difference in BMI was observed in the present cohort. This homogeneity in BMI distribution may be attributable to population selection or uniform patterns of adiposity, thereby diminishing the statistical power to detect sex-based differences. Importantly, the absence of a sex difference in BMI does not negate the role of obesity in KOA pathogenesis; rather, it suggests that fat distribution patterns particularly visceral adiposity may exert a stronger influence on KOA than BMI per se.

CRP, an acute-phase protein synthesised by the liver, is widely employed as a systemic marker of inflammation, with elevated levels indicating heightened inflammatory activity (20). As a chronic degenerative disorder, KOA is frequently accompanied by persistent low-grade inflammation that drives articular cartilage degradation (21). Elevated CRP may therefore reflect the inflammatory milieu underlying these pathological processes. Although CRP levels did not differ significantly between sexes, the correlation between CRP and K–L grade was numerically stronger in women (*r* = 0.60 vs. *r* = 0.51 in men). The majority of female participants (mean age 65.22 ± 11.01 years) were postmenopausal, with concomitant declines in oestrogen and its anti-inflammatory effects, potentially amplifying the association between CRP and structural damage. Concurrently, reduced oestrogen may disinhibit CRP synthesis and augment inflammatory responses, thereby accentuating the observed relationship. However, this hypothesis remains speculative and requires further investigation.

Among the 258 patients, radiographic K–L grading was distributed as follows: grade 1, 8 (3.1%); grade 2, 72 (27.9%); grade 3, 112 (43.4%); and grade 4, 66 (25.6%). In clinical practice, age is traditionally regarded as a surrogate for disease severity and joint degeneration, whereas BMI is linked to both mechanical loading and systemic inflammation. However, our multivariable analyses revealed that CRP exhibited the strongest conditional association with K–L grade among the included predictors, with a standardized regression coefficient exceeding those of BMI and age. This observation is consistent with previous reports (7, 22) that identified CRP as a quantifiable indicator of intra-articular inflammation predictive of radiographic severity. Although Kondo et al. (8) reported an association between elevated baseline CRP and subsequent radiographic progression in a community-based cohort, our study is among the first to validate this association in an Asian clinical population. These data suggest that inflammatory mechanisms may play a more dominant role in KOA severity than traditional mechanical stress (BMI) or age-related degenerative processes, supporting the incorporation of CRP -a readily available and cost-effective inflammatory biomarker into clinical algorithms for risk stratification. While these findings do not directly warrant changes to clinical guidelines, they support further investigation into the role of inflammation in KOA severity.

ROC analyses yielded good discriminative performance for the combined model in both males (AUC = 0.865) and females (AUC = 0.880), underscoring its practical utility for identifying patients with moderate-to-severe KOA within the current sample. Current clinical practice guidelines from the American Academy of Orthopaedic Surgeons (23) recommend conservative management (exercise, weight reduction, pharmacotherapy) for mild KOA, whereas joint replacement is considered the standard of care for moderate-to-severe disease. Consequently, the derived risk-prediction model may inform clinical decision-making regarding the optimal timing of surgical intervention.

The discordance between the identified factor weights and those of traditional risk factors highlights the multifactorial complexity of KOA severity and emphasises the necessity of comprehensive patient assessment that transcends conventional parameters. These findings suggest a need to re-evaluate the weighting of established risk factors within clinical algorithms and guidelines, advocating for multidimensional assessment encompassing both biological and psychosocial domains.

### Limitations

Several limitations warrant consideration. First, the retrospective, cross-sectional design precludes causal inferences; although CRP correlates with KOA severity, prospective studies are required to delineate causality. Second, the sex imbalance in the sample may limit the generalisability of sex-specific findings; future investigations should recruit more balanced cohorts to re-examine sex differences in KOA severity. Third, the study could not account for all potential confounding variables such as joint injury, physical activity, smoking, or comorbidities due to data availability. Furthermore, lifestyle and occupational factors such as physical activity levels, footwear habits, and occupational joint loading were not systematically recorded in the medical records and thus could not be analyzed. Future studies should incorporate these variables to provide a more comprehensive risk assessment. Although magnetic resonance imaging (MRI) offers superior sensitivity and specificity for cartilage evaluation, only x-ray data were analysed for cost-effectiveness and clinical practicality; integration of advanced imaging modalities in future work may yield more comprehensive insights.

## Conclusion

In this cross-sectional study, CRP showed a strong association with KOA radiographic severity after adjusting for age, BMI, and pain scores. A numerically stronger association was observed in female patients, though this difference requires further confirmation. These findings provide a new direction for precision medicine, supporting the further investigation of inflammatory biomarkers into clinical assessment and management strategies to improve patient outcomes. Prospective longitudinal studies are warranted to confirm these observations and establish causality.

## Data Availability

The raw data supporting the conclusions of this article will be made available by the authors, without undue reservation.
